# Synoptic reporting by summarizing cancer pathology reports using large language models

**DOI:** 10.1038/s44401-025-00013-8

**Published:** 2025-04-01

**Authors:** Sivaraman Rajaganapathy, Shaika Chowdhury, Xiaodi Li, Vincent Buchner, Zhe He, Rui Zhang, Xiaoqian Jiang, Ping Yang, James R. Cerhan, Nansu Zong

**Affiliations:** 1 Department of Artificial Intelligence and Informatics, Mayo Clinic, Rochester, MN, USA.; 2 School of Information, Florida State University, Tallahassee, FL, USA.; 3 Division of Computational Health Sciences, University of Minnesota, Minneapolis, MN, USA.; 4 Department of Health Data Science and Artificial Intelligence, UTHealth, Houston, TX, USA.; 5 Department of Quantitative Health Sciences, Mayo Clinic, Rochester, MN, USA.

## Abstract

Synoptic reporting, the documenting of clinical information in a structured manner, enhances patient care by improving accuracy, readability, and report completeness, but imposes significant administrative burdens on physicians. The potential of Large Language Models (LLMs) for automating synoptic reporting remains underexplored. In this study, we explore state-of-the-art LLMs for automatic synoptic reporting, using 7774 pathology reports from 8 cancer types, paired with physician annotated synoptic reports from the Mayo Clinic EHR. We developed a comprehensive automation framework, combining state-of-the-art LLMs, incorporating parameter-efficient optimization, scalable prompt templates, and robust evaluation strategies. We validate our results on both internal and external data, ensuring alignment with pathologist responses. Using our framework, fine-tuned LLAMA-2 achieved BERT F1 scores above 0.86 across all data elements and exceeding 0.94 over 50% (11 of 22) of the data elements, translating to manually assessed mean semantic accuracies of 77% and up to 81% for short clinical reports.

Synoptic reporting is the process of formatting narrative pathology reports into a specific structured format^1^. The College of American Pathologists (CAP) has promoted the use of synoptic reporting since 1998, due to a number of advantages for various stakeholders in cancer diagnosis and treatment^1,2^. The standardized presentation, improved completeness, and conciseness enable clinicians to access pertinent information with greater ease and speed^1,3–5^. Through improved interoperability, synoptic reports also enable data registrars to build large databases for quality control, public health reporting, and research^2,6^. Most importantly, they encourage compliance to standards of care and accurate exchange of vital information, improving patient care and potentially patient outcomes^7,8^.

The CAP defines the synoptic reports as consisting of *data elements* and corresponding *element responses*^9^ that capture the information present in an unorganized narrative text^6^. Documentation and administrative work has been shown to take 22% of a clinician’s work day on average^10^. The writing of reports in a highly engineered synoptic fashion is known to further add to this high administrative demand^6,11,12^. In addition, the transcription error rates have been positively correlated with the number of unique data elements required^1,3^. The conversion of existing narrative reports for research is yet another source of burden. To leverage the advantages while navigating these challenges necessitates automated synoptic reporting.

Existing work on automating synoptic reporting is limited in scope. The techniques from open information extraction, knowledge engineering, and natural language processing (NLP) are combined using domain expertize for structurizing pathology reports in refs. 13–15. A heuristic search based approach is proposed in^16^ to process semi-structured pathology reports. These proposed heuristic methods require extensive redevelopment of the algorithms for pathology reports from a new source^13,14,16^. Lam et al.^17^ described a rule-based NLP system to convert a semi-structured extensible markup language (XML) document into more structured tabular data. How such a system can be adapted for processing unstructured reports was not made clear. Some authors proposed a report-level classification approach^18,19^. Wu et al.^18^ suggested the use of a graph convolutional network and Alawad et al.^19^ proposed the use of a parameter-sharing multi-task convolutional neural network for classifying the reports. Such a classification approach is only appropriate for handling data elements whose responses can be chosen from a set of finite categories, such as cancer stage or type. However, report-level classification is unsuitable for data elements whose responses are not categorical, such as the size of a tumor or the mitotic rate. Mu and colleagues^20^ demonstrated that a bidirectional encoder representations from transformers (BERT) trained using a binary relevance (BR) method can analyze textual pathology synopses and map patients to diagnostic keywords. The BR method converts multiple semantic labels into multiple binary predictions. The authors reported that this transformation resulted in loss of information about the correlations between labels^20^. Thus the model could not exploit the fact that some labels often occur with others^20^. It can be seen that the existing state-of-the-art NLP-based methods for extracting structured data from unstructured data suffer from being limited in the types of reports they can analyze^15–17,21^, have the tendency to lose important semantic information^14^, require semi-structured data^17^, or require large amounts of expensive expert labeled data^21^.

Large Language Models (LLMs), a recent advancement in artificial intelligence, offer a potential method to overcome the limitations of NLP-based approach to synoptic reporting. LLMs are large arrays of deep neural networks that are trained on massive corpora of text data^22^. The training of the models is typically done in a semi-supervised fashion, wherein, only a small amount of the data needs to be adjudicated and annotated by human experts^23^. This enables LLMs to leverage the knowledge contained in large databases in a more cost-effective manner, compared to traditional NLP methods^24^. The training of large models with large text data has been shown to result in emergent abilities^25^. Here, an emergent ability is a capability that is observed in a large version of a model but is not present and could not be extrapolated from the capabilities of smaller versions of the model^25^. These key advantages have triggered studies on evaluating the effectiveness of LLMs in the clinical setting, such as clinical text annotation^24^, medical question and answering^26^, and reasoning in the medical domain^27^—which form essential components of synoptic reporting. Indeed, prompt-based use of OpenAI’s ChatGPT, with the LLM GPT-4, is proposed to fill out synoptic reports in^28,29^. Inferences from ChatGPT require sharing the pathology reports with OpenAI^30^. This limits the approach since we need to protect patient health information and data privacy. While LLMs offer a promising path for automatic synoptic reporting, inaccuracies due to fabricated facts and unverified training data, lack of interpretability of the results, and potential risk of security breaches are some major challenges^23,30^.

In this work, we build a proof of concept, flexible framework, which can leverage a variety of LLMs for synthesizing synoptic reports from unstructured pathology reports. This framework integrates innovative prompt engineering and parameter-efficient fine-tuning strategies to create LLMs that produce synoptic reports that align with pathologist-created synoptic reports. We test the models rigorously using the state-of-the-art BERT F1 scores, augmented by manual validation to ensure semantic accuracy and clinical applicability. To ensure that our results are robust to changes in the source and formatting of the reports, we validate our models on a sample of external data. We demonstrate a pathway for automating synoptic reporting from narrative cancer pathology reports using LLMs while providing comprehensive insights into their strengths and weaknesses for this task.

## Results

### Data

We digitally extracted 7774 cancer pathology reports of 7228 unique patients from the Mayo Clinic’s Unified Data Platform (UDP)^31^. The pathology reports consist of four components: 1) the gross description, 2) the preliminary frozen section consultation, 3) the diagnosis, and 4) the synoptic report (see Supplementary Note 1 for details). The first three components (1–3) are written by a pathologist in a natural language format or using a partially organized format (e.g., sections, lists), without strictly following a standardized predefined schema. From here on, we refer to the combination the gross description, the preliminary frozen section consultation, and the diagnosis as the “*unstructured report*“. These synoptic reports are in use for patient care at the Mayo Clinic, hence we treat these reports as the gold standard reference for model development and evaluation. The synoptic report consists of several *data elements* and corresponding *element responses* (e.g., *Specimen Laterality: Right*). The exact data elements included in each report vary. In this study, we have utilized the top 22 most frequently occurring data elements. The summary of the data is shown in Table 1.

### Proposed framework—LLM based pathologist aligned automatic synoptic reporting

**Fig. 1 |:**
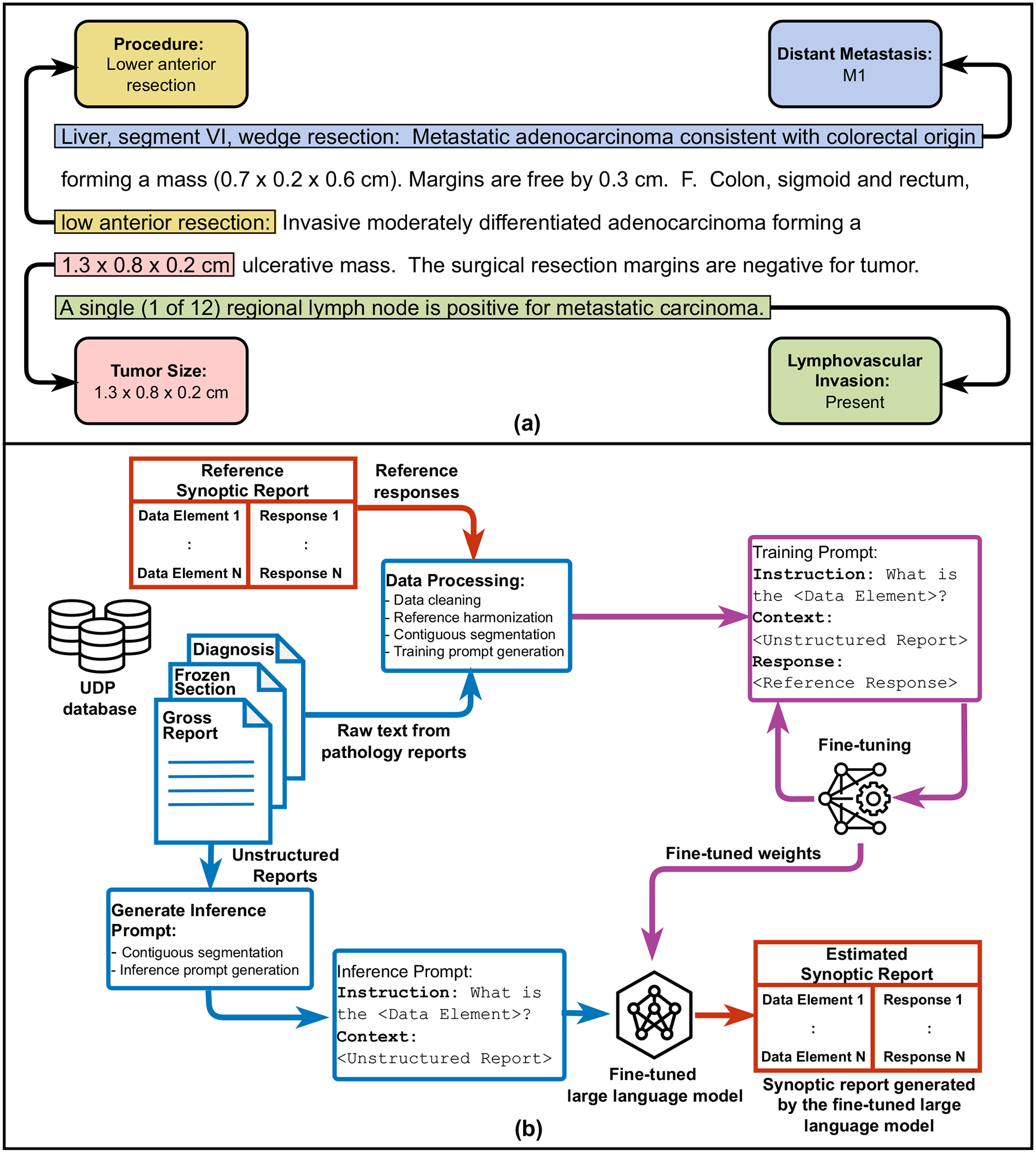
Overview of the proposed LLM based automatic synoptic reporting framework. **a** An excerpt of a de-identified sample pathology report is shown. The boxes show example data elements along with the corresponding data element responses as reported by Mayo Clinic pathologists. Here “Procedure”, “Tumor Size” are NLP type data elements, and “Distant Metastasis”, “Lymphovascular Invasion” are classification type data elements. The text highlights show the most relevant region of the report where the information for the response is located. **b** To generate a synoptic report from an unstructured pathology report, we take an element-by-element, prompt-based approach. The unstructured sections of the pathology report are combined, cleaned, and processed to create training prompts. Training prompts consist of the instruction, unstructured report, and reference response concatenated. The model is fine-tuned on the training prompts and learns to associate the specific reference response with the unstructured report and given instruction. To obtain an inference from the model, an inference prompt is created that is identical to the training prompt except it does not contain the reference response. The model is given only the instruction and the unstructured report during inference and is expected generate response text that follows the inference prompt.

The synoptic reporting task we endeavor to automate is described as follows. Given an *unstructured report*, we utilize an LLM to generate a corresponding synoptic report with pre-specified data elements and element responses. We refer to the data element responses generated by an LLM as ‘*estimated element responses*.’ We refer to the data element responses filled in by the pathologist in the original data’s synoptic reports as ‘*reference element responses*.’ Relevant example sections of the unstructured reports along with sample data elements and corresponding element responses, as entered by Mayo Clinic pathologists, are shown in Fig. 1a. The highlights indicate the region of the report where the most relevant information is available. Note that some data element responses could be grouped to a pre-defined finite set of categories that are seen both in the training and test data. We refer to these as *classification type* data elements. In Fig. 1a, “Distant Metastasis“ and “Lymphovascular Invasion” are classification-type data elements. For example, the responses to the data element lymphovascular invasion could be grouped to one of the following: present, intermediate, absent, result pending, or other. When such a grouping of the element responses is not possible, we refer to them as *NLP type* data elements. The data elements “Procedure” and “Tumor Size” shown in Fig. 1a, are examples of NLP type data elements. The tumor size is an NLP type data element, since the numeric responses could take on a wide range of values and a finite grouping would result in significant information loss in the answer. In Supplementary Table 1, we show how we identify each of our data elements as either classification or NLP type.

To automate the synoptic report synthesis using LLMs, we employ a prompt-based^32^, element-by-element approach. An overview of this strategy is shown in Fig. 1b. We describe this strategy in more detail as follows.

### Comparison with baselines of alternative approaches

While we primarily focus on the use of generative LLMs to automate synoptic reporting, here we compare the performance of our best-performing fine-tuned LLM with baseline models that propose solutions for cancer synoptic reporting using alternative approaches. In Supplementary Table 3, we summarize studies that have proposed different approaches for automating synoptic reporting. We observed that studies that use a heuristic search technique assume semi-structured data and are not applicable for our study where we assume starting from fully unstructured pathology reports. Other broad strategies excluding LLMs, are report-level classification and named entity recognition (NER). In report-level classification, the model assigns the entire unstructured report a predefined category based on the contents of the report. In NER, the model categorizes each word in a sentence as either belonging or not belonging to a predefined set of categories or named entities. Out of the studies that utilize a report-level classification approach or an NER approach, very frequently we were not able to obtain the model or model weights that are essential for an experimental comparison. Further, for the NER approach, if the original annotated data are not shared, we are unable to train on our own data. Doing so would require a fine-grained word-level expert annotation of our data, which is infeasible. The studies we are able to compare experimentally are the hard parameter sharing MT-CNN model^19^ and the CancerBERT^33^ model for automatic synoptic reporting to provide additional baselines that do not use a generative LLM (see Supplementary Note 4 for experimental details).

**Fig. 2 |:**
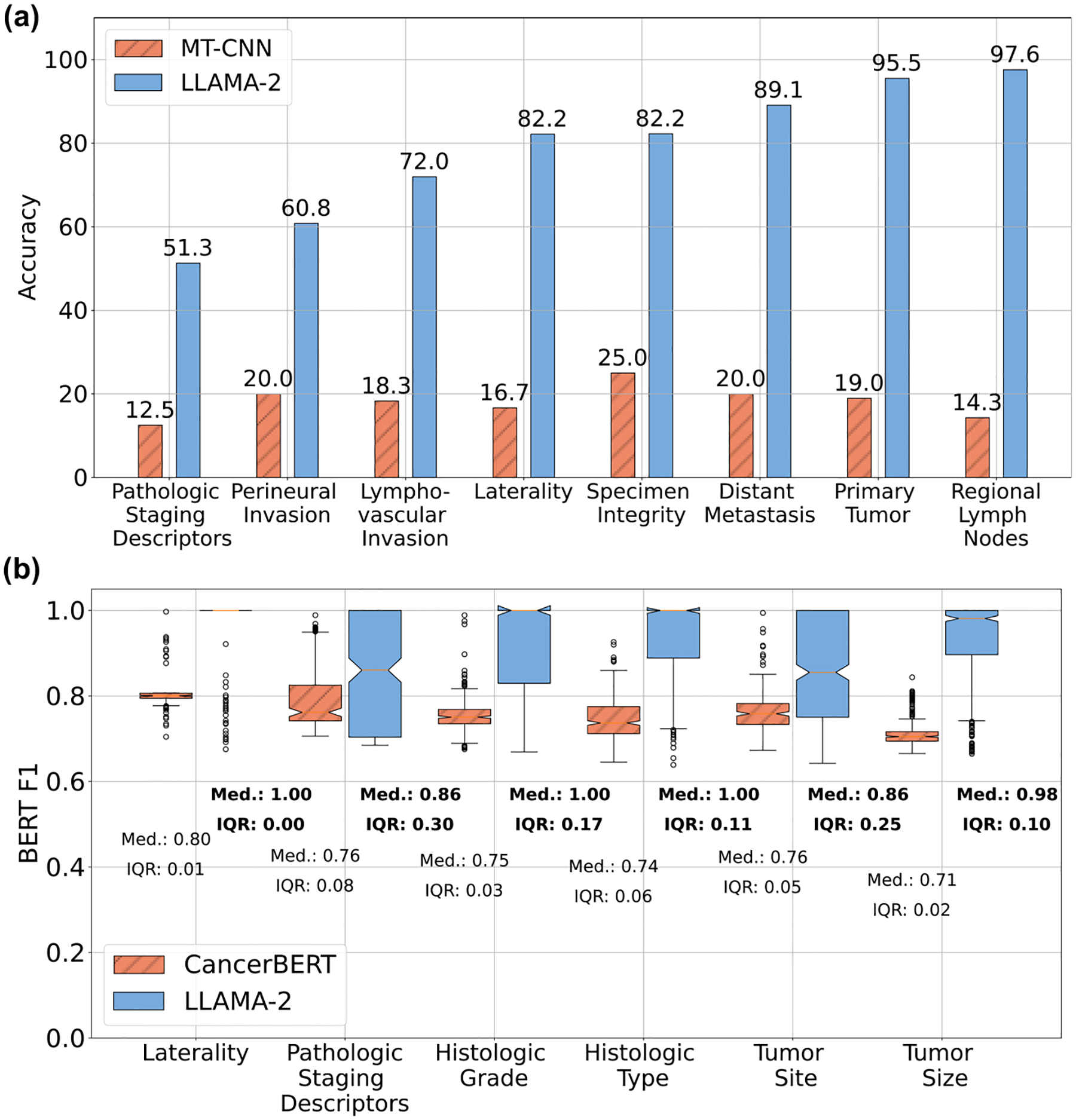
Comparative performance with alternate approaches. **a** Performance comparison of the fine-tuned LLAMA-2 generative model with the MT-CNN model that uses a report-level classification approach. **b** Performance comparison of the fine-tuned LLAMA-2 generative model with the CancerBERT model that utilizes a sentence-level classification approach (also known as NER).

We present the performance of baseline models that take use alternative approaches to creating synoptic reports, in Fig. 2. In Fig. 2a we observe that the class-balanced accuracy of MT-CNN, which uses report level classification, ranges from 12.5 to 25%, while the accuracy of the fine-tuned LLAMA-2 ranges from 51.3 to 97.6%. We use the balanced accuracy instead of BERT F1 score to be more fair to the report-level classification-based MT-CNN since its output can only vary between one of the several pre-defined categorical values for a given data element. We show by comparison that using LLMs in the generative mode is a promising approach to automating synoptic reporting when compared to the classification or NLP-based approach. We note that the poorer performance of the MT-CNN is likely due to the smaller size of our dataset—we have a total of 7774 pathology reports while the authors in^19^ have 71,223 reports available for model development.

In Fig. 2b we observe that the CancerBERT model^33^ that proposes an NER approach has BERT F1 scores in the range of 0.71 to 0.80 in comparison to the fine-tuned LLAMA-2 whose BERT F1 ranges from 0.86 to 1. We underscore that the much higher CancerBERT model performances reported in the original literature^33^ are for the NER task and not for the synoptic reporting task. Importantly, the models using alternate approaches do not provide an end-to-end solution for automating synoptic reporting. The MT-CNN is only suitable for data element responses that can easily be categorized into a set of pre-defined labels. This categorization limits its applicability to a narrow range of data elements and reduces the richness of information that can be presented in a synoptic report. While the NER approach used by the CancerBERT model can be applied to a wider range of data elements, it results in multiple answers for each data element, necessitating additional engineering to identify the most suitable response.

### Out-of-the-box vs fine-tuned models

The generative LLMs models we selected (BERT, GPT-2, LLAMA-2, LLAMA-3) to study have been pre-trained on large corpora of unstructured generalized text data (see Supplementary Note 3 for details). The dataset used for the pre-training is unspecialized and is likely not suited for synoptic reporting. From here on, we refer to these models in the default configuration provided as-is by the model sources as the *out-of-the-box* models^34^. While prompting^32^ has been shown to enable LLMs to perform specialized downstream tasks, only moderate performance has been reported for annotating medical texts^29^. Therefore, we *fine-tune*^35^ the models, wherein the model parameters (or a fraction of the model parameters) are updated through training on relevant medical text, to improve performance on the downstreamtask^36^. We use zero-shot prompting, where only the query and context are sent to the model without any prior examples^32^. We use the zero-shot method to efficiently utilize the limited input token sizes. We use identical prompts for both out-of-the-box and fine-tuned models and compare them on the same test dataset.

**Fig. 3 |:**
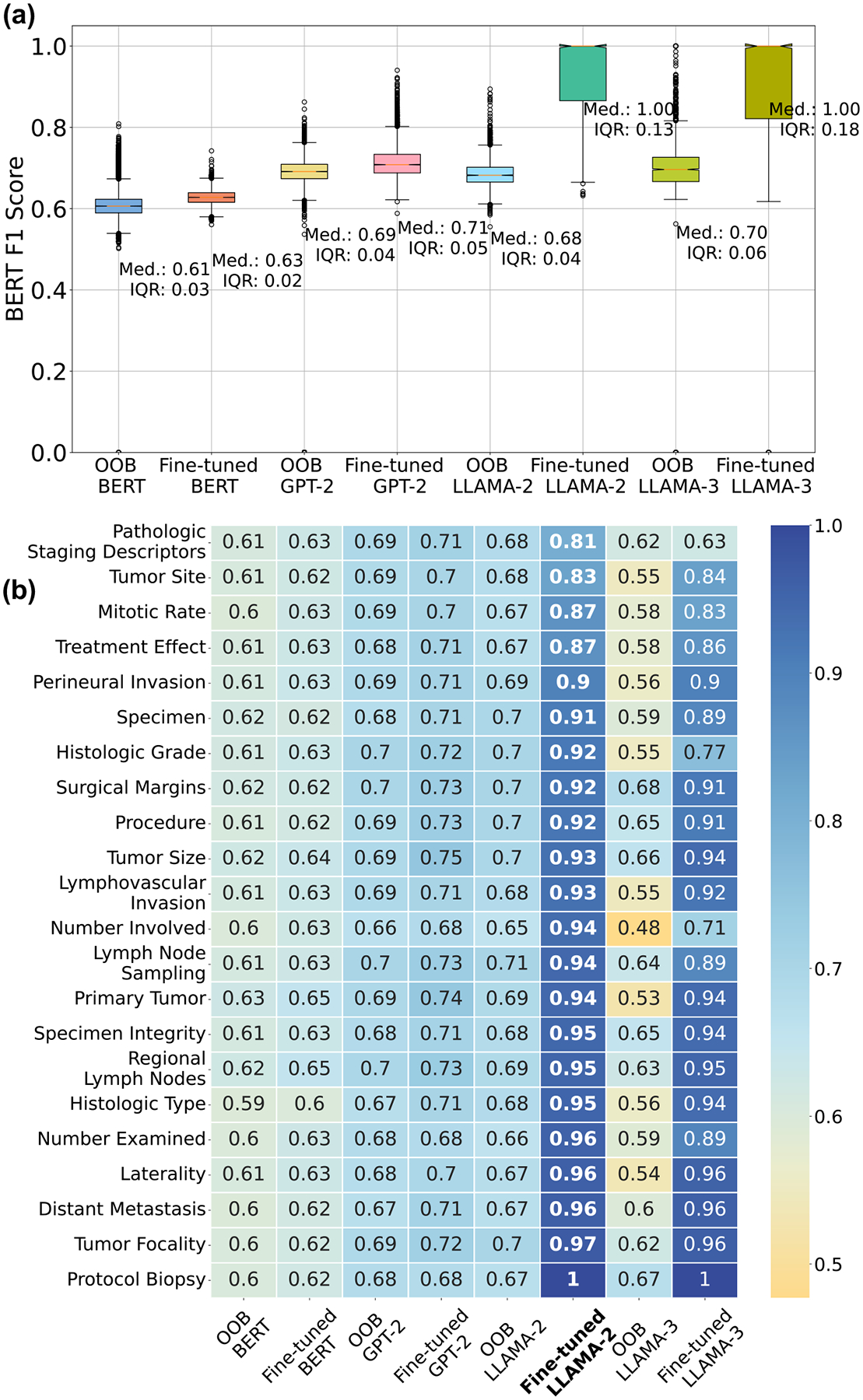
Performance of fine-tuned models versus out-of-box models. **a** Performance comparison of out-of-the-box (OOB)and fine-tuned language models on the synoptic reporting task. A BERT F1 Score of 1 indicates a perfect match between the model’s output and the reference answer. The median and the IQR^37^ are reported in the plots. **b** Performance of the out-of-the-boxand fine-tuned language models, segregated by the data element. The numbers inset in the heat map show the mean BERT F1 score. The color scale of the heat map is shown on the right and represents the BERT F1 score.

Figure 3 compares the performance of the out-of-the-box and fine-tuned models on estimating the data element responses. Figure 3a shows a summary of the BERT F1 scores for the test set composed of 22 data elements. Fine-tuning improves the median of the score, with LLAMA-2 having the largest increase (median of BERT F1 score from 0.68 to 1). The inter quartile range (IQR), which is the difference between the 75th and 25th percentiles of the data^37^ of the fine-tuned LLAMA-2 model increases from 0.04 to 0.13, indicating that the model has a wide range of performances depending on the data element type. This is clarified in Fig. 3b, which shows the detailed stratified performance of the out-of-the-box and fine-tuned models on each data element tested. The numbers inset in the heat map show the mean of the F1 score. The range of scores for the out-of-the-box LLAMA-2 varies from 0.67 to 0.70, while the fine-tuned version ranges from 0.81 (for Pathologic Staging Descriptors) to 1 (for Protocol Biopsy).

The use of prompting on its own, with the out-of-the-boxmodels does not yield accurate synoptic reports, with resulting BERT F1 scores ranging from 0.61 to 0.70 (see Fig. 3a). Fine-tuning improves the performance, with greater improvement obtained in proportion to the size of the model. Indeed, the LLAMA-2 variant with 7 billion parameters after fine-tuning is able to achieve median BERT F1 scores of 1.00 (IQR = 0.13) from 0.68 (IQR = 0.04) for its out-of-the-boxvariant, as shown in Fig. 3a. This observation of a sudden significant improvement as the model size increases by 2 orders of magnitude corresponds well with the property of emergent behavior reported in LLMs^25^. We focus the analysis on the best performing model (LLAMA-2) from this experiment onwards.

### Performance across cancer types

**Fig. 4 |:**
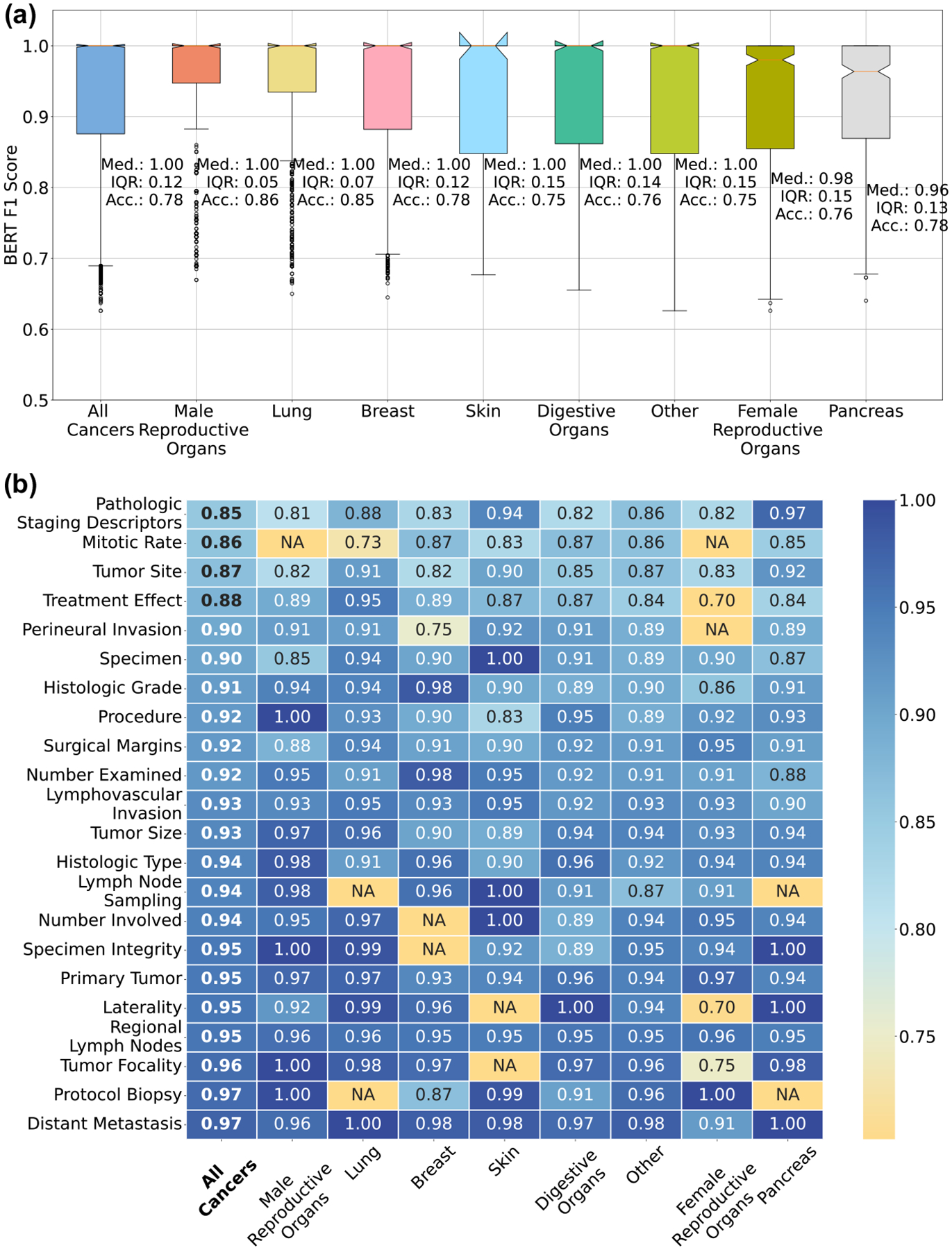
Performance across cancer types. **a** Performance comparison of fine-tuned LLAMA-2 model on different cancer types. **b** Detailed view of the performance across cancer types for each data element type. The boxes labeled ‘NA’ indicate cases where the specified data elements are missing in the reference synoptic reports for the cancer type.

Here we compare the performance of the fine-tuned LLAMA-2 model on reports describing the cancer categories shown in Table 1. This is done to check if the model performance is strongly influenced by the type of cancer being described in the unstructured report. We use the primary diagnosis of cancer to categorize the unstructured reports. We show the performance of the fine-tuned LLAMA-2 model on unstructured reports stratified by the primary cancer diagnosis the report describes in Fig. 4. The box plots in Fig. 4a show the summary of the BERT F1 scores for all non-missing data elements in the unstructured reports for each cancer type. The median of the scores ranges from 0.91 to 0.95 for the different cancer types. Figure 4b shows the detailed breakdown of the model performance for each non-missing data element for the different cancer types.

Our results indicate that for the cancer categories represented in our dataset, the fine-tuned LLAMA-2 model can effectively create synoptic reports without the need for prior knowledge about the type of cancer being described in the report. Such a feature is useful since the diagnosis of the cancer type will not always be available. Indeed, the gross report is often written before a conclusive diagnosis has been reached. An interim synoptic report derived from the clinical notes before diagnosis is complete could offer many of the same benefits as one created after diagnosis.

### Size effects: reports, fine-tuning data, and model sizes

It can be seen from Supplementary Table 2, that the LLMs have strict limits on the number of words they can process in an input. A significant number (~ 56% for LLAMA-2) of our unstructured reports exceed the input limits of even the LLAMA-2 model with a capacity of 4096 tokens. We handle such large reports by segmenting them into smaller contiguous pieces so that each model has an opportunity to scan the whole unstructured report. The best-estimated data response amongst the estimates obtained from each piece is selected for evaluation. Further, we quantify the amount of improvement capacity left in the fine-tuned models. We do this by varying the dataset size used for fine-tuning and reporting the test performances of the partially fine-tuned models. We also test two variants of the LLAMA-2 model – the 7 billion and 13 billion parameter variants.

**Fig. 5 |:**
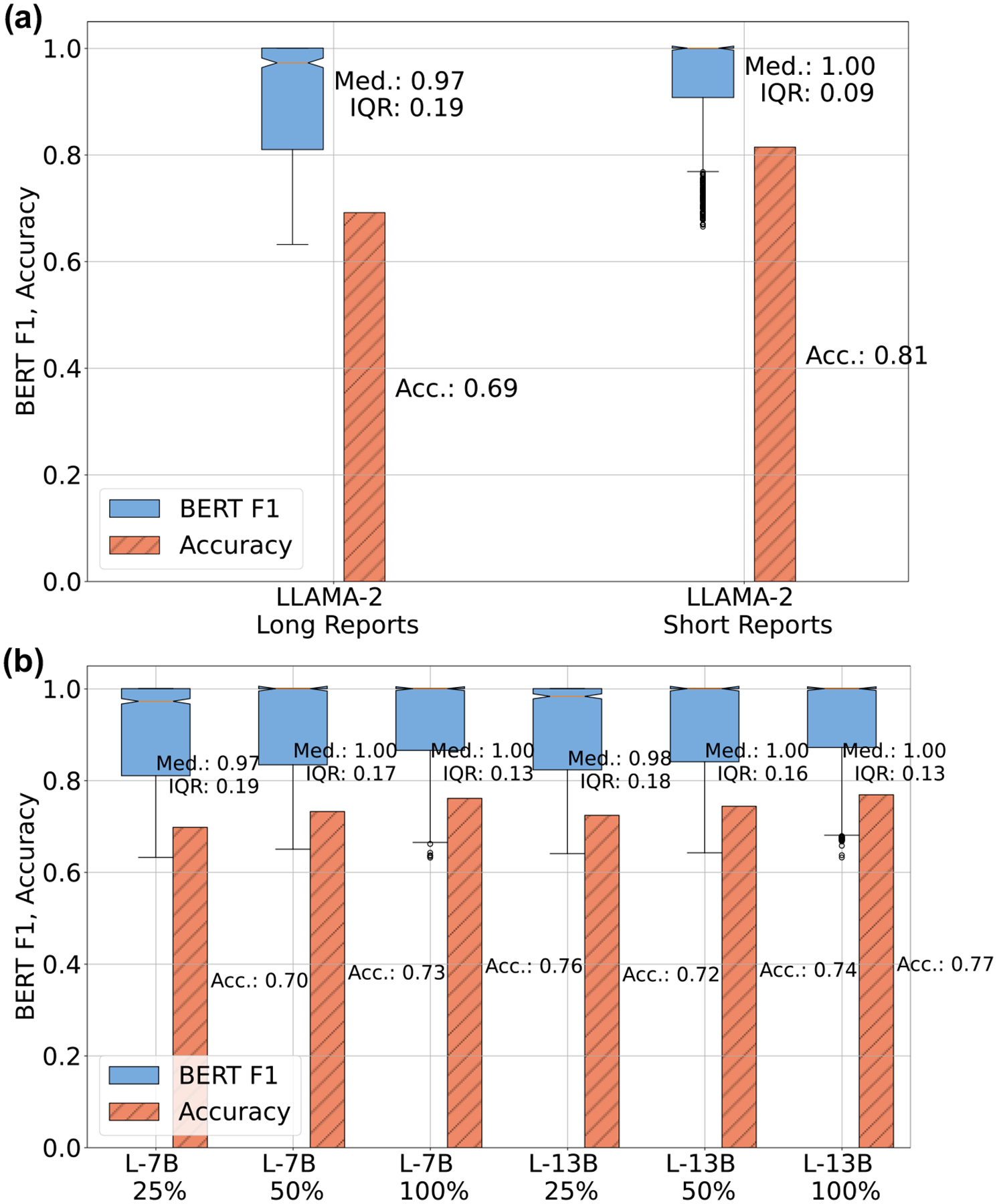
Size effects in LLM based synoptic reporting. **a** Performance differences in the fine-tuned LLAMA-2 model with variation in the size of the clinical notes. Long reports are clinical notes whose size (in no. of words) exceeds the token limit (4096), whereas short reports are clinical notes whose size (in no. of words) is less than the model’s token limit. Long reports are broken into smaller pieces to fit the model’s token limit, leading to its inability to answer questions accurately and a reduction in performance. The projected accuracy is computed using a threshold of BERT F1 > 0.85 to closely align with manual analysis. **b** Performance comparison of fine-tuned LLAMA-2 models on the synoptic reporting task as the size of the training data is increased. Two variants of the LLAMA-2 model are tested, the 7 billion parameter version (L-7B) and the 13 billion parameter version (L-13B).

The contiguous segmentation we use to solve the input token limits reduces the field of view of the models. In Fig. 5a, we show the performance degradation of fine-tuned LLAMA-2 due to the reduction in the field of view. When the complete unstructured report fits in the field of view of the model, its performance is improved: the median BERT F1 scores increase to 1 (IQR = 0.09) from 0.97 (IQR = 0.09). The projected accuracy scores show an improvement of 12 percentage points (81% for short reports vs 69% for long reports). In Fig. 5b, we present the gain in performance we can achieve if the size of the training data is increased. We observe a small improvement in the performance, with the projected accuracy changing from 0.70 to 0.76 when the size of the training data is increased from 25% to 100%. An increase in the parameter size by roughly 50% from 7 billion to 13 billion does not produce a corresponding improvement in performance: the 13 billion parameter variant has accuracies that are 1 to 2 percent points higher than its 7 billion parameter variants.

### External validation

Sushil et al.^29^ provide a sampling of oncology reports that have been curated and annotated by experts, for the purpose of testing the performance of language models. We use this data for external validation. The dataset consists of 40 de-identified breast and pancreatic cancer progress notes. Three categories of labels have been given to the text, which include entities, attributes of the entities, and relationships between the entities. We reviewed these labels and determined that only 4 labels under the entities category match well with 4 of 22 data elements we have considered in our analysis. These are Histologic Type, Laterality, Tumor Site, and Procedure. We use our models to extract the responses to these 4 data elements from their reports and compare the model responses to the expert-annotated labels.

**Fig. 6 |:**
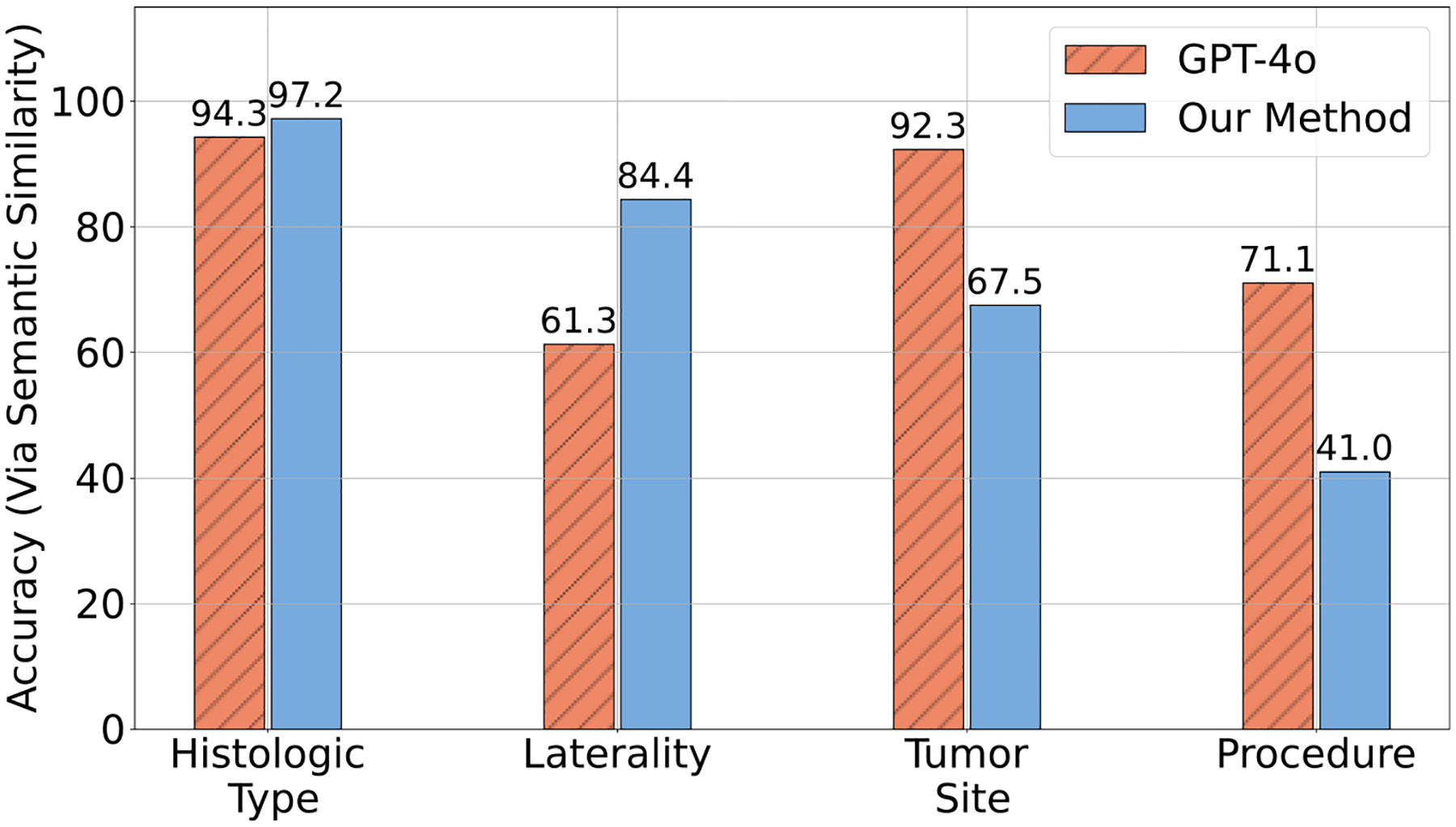
Benchmarking with external validation data. Performance of the OpenAI’s GPT-4o and the fine-tuned LLAMA-2 7B model (best-performing model) on an external dataset in Sushil et al.^29^. We test the ability of the two models to extract 4 data elements which correspond to the labels provided in the external dataset. The accuracy reported here is obtained by manual comparison of the labels provided in ref. 29 against the model responses. *Note: Fine-tuned LLAMA-2 has only been fine-tuned on our data, it has not seen examples from the external data*.

In Fig. 6, we compare the performances of our proposed fine-tuned LLAMA-2 7B model and OpenAI’s GPT-4o^38^ model on the external, expert-annotated data from Sushil et al.^29^. We employ GPT-4o in the zero-shot setting, to provide an objective out-of-the-box comparison with our proposed method. We manually compare the responses given by the models for semantic similarity, using the labels provided in the external dataset. The fine-tuned LLAMA-2 model described here is the same as the model described in earlier parts of this study—it has only been fine-tuned on our data; it has never been shown any examples from the external data. We observe that the fine-tuned LLAMA 2 outperforms the GPT-4o model on two data elements—the Histologic Type and Laterality, while GPT-4o outperforms our method on identifying the Tumor Site and Procedure.

## Discussion

In this study, we showcase using LLMs to automatically generate synoptic reporting that includes 22 data elements, with an element-by-element prompting strategy, across multiple cancer types. Our strategy leverages existing synoptic reports, thus alleviating the need for expensive, dedicated expert text annotations. Additionally, the use of a universal prompt template for each data element, enables our strategy to be scalable to a larger set of data elements that may be needed in a clinical setting.

The experiments in this study show that fine-tuned generative LLMs, specifically LLAMA-2/3, significantly outperform out-of-the-boxmodels as well as models that use classification or NER (see Figs. 2 and 3). The use of fine-tuning and a universal prompt template allowed our models to handle diverse data elements, including those that involve classification as well as those which require NLP. Our approach achieved BERT F1 scores as high as 0.94 for more than half the data elements (shown in Fig. 3b). This translates to an average accuracy of 77%, and up to 81% for short pathology reports that fit within the input context windows of the LLMs. In addition, our best performing fine-tuned LLM is agnostic to the cancer being described in the unstructured clinical note, making it useful when the diagnosis of cancer is not available a-priori (see Fig. 4). We further show that our technique is generalizable, with validation performance on unseen external data at par with GPT-4o (shown in Fig. 6). Our framework supports a flexible integration of diverse LLM models and enables rapid deployment on institutional hardware, wherein all patient data used for training and testing are stored locally. As a proof of concept, we highlight the findings and issues raised in this study.

We utilize open-source models that can be trained and stored entirely locally, within the institutional hardware. This enables us to avoid sharing sensitive patient health information with a third party LLM services as would be required in a cloud based LLM such as OpenAI’s GPT-4o^30,38^. Some closed-source LLM vendors offer services compliant with the Health Insurance Portability and Accountability Act (HIPAA). However, due to the rapid advancement in AI, many challenges remain open in safeguarding patient privacy even with HIPAA-compliant LLMs^39,40^. As a result, not all hospital systems may have a contract with a closed-source LLM vendor that protects patient information. The inability to download and share a fine-tuned, clinically validated model is another major limitation of many closed-source LLMs^30^. Our approach of using open-source LLMs thus promotes model sharing, transparency, and further research. Moreover, the strategy of using local models ensures they are protected by institutional firewalls. The needs-based access within the institution limits opportunities of sensitive data leakage through adversarial inference attacks^41,42^. At the time of writing this report, we have shared our methodology and code for adoption in other hospital settings. In summary, open-source LLMs provide key advantages, including robust privacy by keeping models and patient data within institutional boundaries, certified deployment through transparent architecture, and predictable inference costs. They also enable reproducibility and further research through model sharing. On the other hand, closed-source, cloud-based LLMs such as GPT-4o, offer benefits such as frequent updates, larger input token limits, and reduced initial infrastructure investments. After evaluating the needs of our study, we adopted the open-source approach to ensure alignment with our priorities of privacy, transparency, and adoptability for future research.

**Fig. 7 |:**
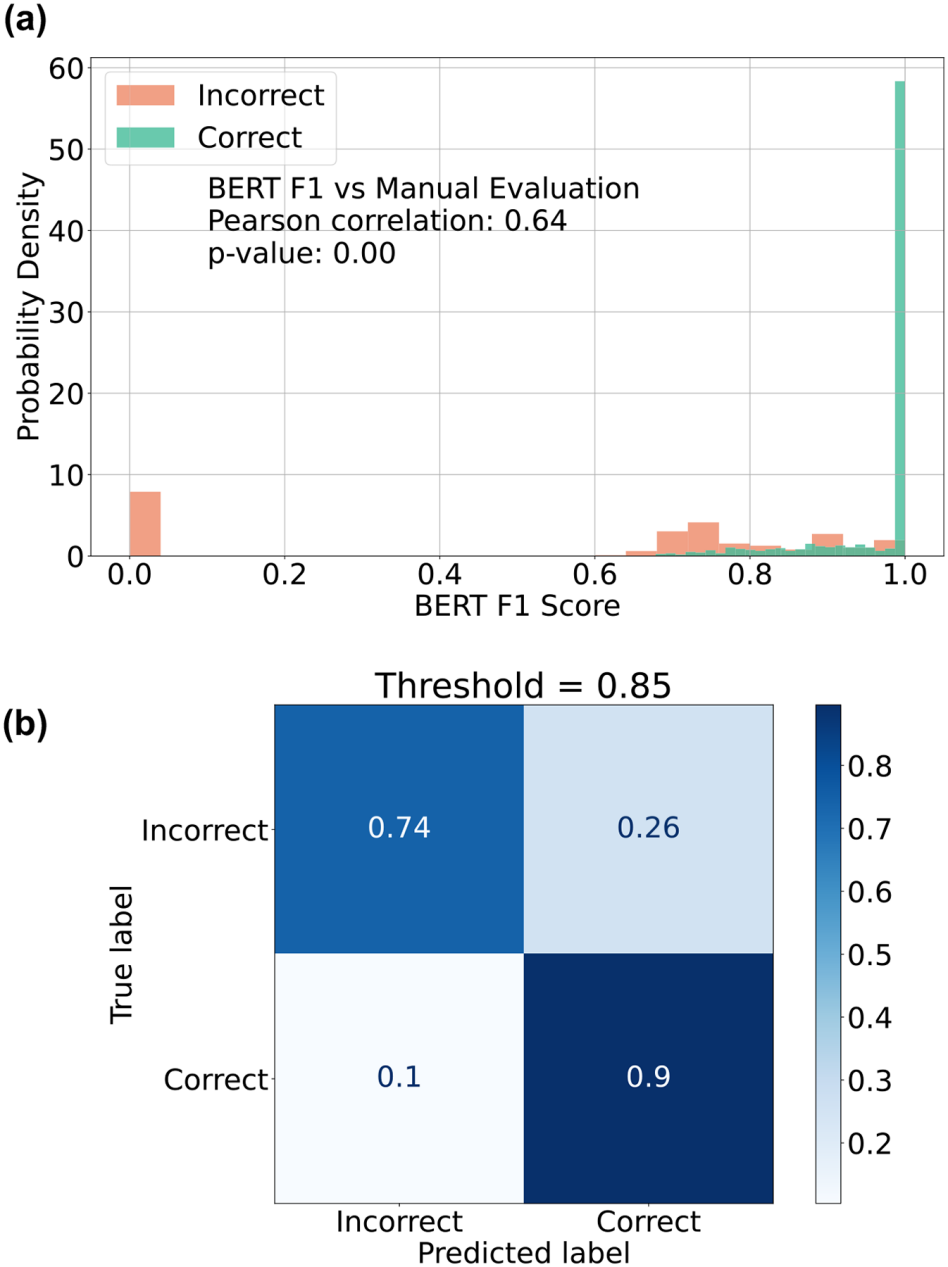
Evaluation strategy—projected accuracy from BERT F1 Score. In (**a**), we show the distribution of BERT F1 scores obtained by comparing the reference element responses and the estimated element responses for the LLAMA-2–7B model. A test set of 100 examples for 22 data elements were used. The correctness and incorrectness of a response were determined by manual comparison. The Pearson correlation between the manual score and BERT F1 is 0.64 and the *p*-value of 0 indicates the correlation is significant. **b** The confusion matrix for BERT F1 score-based determination of the correctness of a response—a threshold of 0.85 is used in this study.

The implications of the use of BERT F1 scores warrant further discussion. The output of our generative LLMs is text responses that have been engineered to mimic pathologist-created synoptic reports. Consequently, when we evaluate our model responses using the pathologist-created reports as a reference, using exact match as a performance metric leads to a significant underestimation of performance. On the other hand, employing simple text-matching metrics risks overlooking semantic information and potentially overestimating model accuracies. We show that the BERT F1 correlates well with manually derived semantic accuracy (Pearson coefficient = 0.64). The BERT F1 scores can then be translated to accuracies using a threshold of 0.85. This BERT F1-derived accuracy aligns with manual evaluation (see Fig. 7).

We acknowledge the following limitations to ensure the scope of our study is well-defined. 1. Model input capacity: We have demonstrated that our system’s performance is negatively impacted by large reports whose sizes exceed the input token limit of an LLM. To address this limitation, we have used segmenting, i.e., splitting the reports into contiguous pieces that fit within the input capacity. While segmenting is an effective intermediate solution, such splitting reduces the context window and increases the risk that the model misses critical information. Recent advances in LLM architectures have enabled significantly expanded token limits (e.g., LLAMA-3’s input token limit of 8196 vs LLAMA-2’s limit of 4096). Such enhanced capabilities will allow models to process longer reports in their entirety, reducing the need as well as impact of segmentation. Our proposed approach serves as a practical stop-gap solution that ensures robust performance within the constraints of the current generation of open-source LLMs while remaining adaptable to leverage improvements of future generation of LLMs. Future research could explore strategies that compress the data without sacrificing critical details, optimizing performance for both the current and future LLMs. 2. Testing GPT-4o on Internal Data: Due to strict organizational policies on protecting patient health information, we were unable to test GPT-4o directly on internal Mayo Clinic pathology reports. While we included a comparison of GPT-4o with our proposed method on external, expert-annotated data, this may not accurately represent the model’s performance on internal reports. Future validation studies could explore institutional collaborations or simulated data to facilitate such comparison while adhering to data security and privacy policies.

3. Breadth of training dataset: Due to the source of the data used, our study and observations are largely restricted to the writing styles utilized by clinicians at the Mayo Clinic. While we provide results (see Fig. 6) from validation on one publicly available dataset external to the Mayo Clinic, it is restricted to four data elements, 2 cancer types, and no additional fine-tuning. Expanding the training dataset to include synoptic reports from other hospital systems and from clinicians whose first language is not English should improve robustness to writing style differences. Additionally, our objective was to create synoptic reports that follow the template used by the Mayo Clinic physicians, which, while guided by the CAP does not necessarily strictly adhere to it. An expansion of the dataset could also improve the model performances that is adaptable to differences in institutional templates. 4. Fine-grained annotations: We used data that is used in practice as-is with data element responses provided by Mayo Clinic pathologists, which we treat as the gold standard reference, against which we can compare the model responses. This level of annotations is sufficient for automating our task and enables scalability and rapid prototyping. However, creating a benchmark dataset incorporating high-resolution annotations within the unstructured pathology reports could enable improved analysis of performances on nuanced cases and increased trust in the results needed for adoption of the proposed technology by clinicians. 5. Focus on Predefined Data Elements: We restricted our attention to generating element responses for a predefined set of data elements. A more comprehensive automation strategy would involve training the LLMs to autonomously propose relevant data elements. It is unclear whether a single LLM that both proposes the list, as well as the element responses, would outperform a dedicated LLM that is specialized to propose the list. An alternative approach involves leveraging the CAP guidelines to determine the data elements needed. However, this method relies upon the confirmed cancer type diagnosis as the CAP guidelines are tailored to specific cancer types. 6. Limited Multimodal Integration: Our framework focuses on textual data. Integration of multimodal data such as clinical images and genetic test data are not addressed in this study. 7. Scope of disease categories: Our study uses EHRs linked to cancer, wherein synoptic reports are well defined. Broadening the capabilities to include automated synoptic reporting for diseases other than cancers are not addressed in this study.

## Methods

### Data processing and curation

#### Data cleaning and harmonization.

The unstructured as well as the structured components of the raw pathology reports collected from the UDP system, frequently exhibit inconsistencies and require processing before use in our application. The unstructured component of the pathology reports was assessed for completeness. Subsequently, we excluded meta-data associated with patient health information and institutional tracking to retain only the pathologist-authored notes for analysis. The structured component (i.e., the pathologist-authored synoptic reports) was not only assessed for completeness but was further processed along two dimensions. First, the data elements in each report were checked for spelling errors and syntactic variations (e.g., “Tumor Size” represents the same entity as “Size of Tumor” or “Primary Tumor Size”). The data elements were then consolidated into a standard, harmonized list of data elements as shown in Table 1. For the classification type data elements, for use in the classification models, we consolidate and compress the data element responses into a finite list of labels, as shown in Supplementary Table 1.

#### Data permissions.

The data usage was approved by the Mayo Clinic Institutional Review Board (IRB) and was determined to be exempt research.

#### Data splitting.

We randomly allocated 80% of the data for training and the remaining 20% for testing. To ensure our models can generalize to the variabilities in reports for different patients, we ensured that no patient is common to both the training and the test sets. Thus, all of our results are obtained from the response of our models on pathology reports and patients unseen by the models during training.

#### Contiguous segmentation.

The LLMs have a strict limit (shown in Supplementary Table 2) on the number of words they can process, while many of our unstructured reports exceed these length limits. To address this limitation, we split our unstructured reports into appropriately sized contiguous segments to accommodate the limits of each model.

#### Data curation.

We have curated and stored both the raw and processed data in the institutional storage for reproducibility and further research to comply with the institutional review board standards.

### Prompt engineering

We designed a universal prompt template to automate the extraction of any data element and reduce the burden on the users in crafting data element-specific prompts. Using this template, a dedicated prompt is programmatically generated for each data element to maximize the model’s focus on the specified data element and its most relevant context, while avoiding unintentional learning of spurious relationships between different elements and their responses. During training, the prompt includes the reference response to guide the model. The prompts also use special keywords (e.g., “Instruction”) along with a stop sequences (e.g., “###”) to demarcate the different zones of a prompt. In contrast, for inference, an identical prompt without the reference response is used.

### Model fine-tuning and inferences

For fine-tuning an out-of-the-box model, we use training prompts. For fine-tuning, the model’s weights are optimized such that its response matches the reference response. We evaluate the correctness of the estimated element responses by comparing with the reference element responses using the BERT F1 score^43^ (see Supplementary Note 3 for details). We use the Transformers library^44^ to perform the model fine-tuning for BERT and GPT-2, wherein all the weights of the model are updated from the out-of-the-box starting point. For fine-tuning the much larger LLAMA-2/3 models, we adopt the quantized low-rank adaptation (QLoRA), a parameter-efficient fine-tuning strategy^45^. This approach, which only updates a small fraction of the parameters, significantly reduces the computation and memory overhead required for training while achieving performances comparable to training that involves the more expensive full model updates. For all the models, we used the Adam optimizer^46^.

In both our training and inference prompts, we provide the models with a clear and explicit instruction to request the model to produce concise responses based on the context, i.e., the pathology report provided within the prompt. To generate an estimated element response using an LLM, we query it using the inference prompt. By sequentially querying a model for every data element using the inference prompts, we find the estimated element responses to each data element of the synoptic report. The same prompts are used for both out-of-the-box and fine-tuned models. We use the generative models in the causal language mode, wherein the model generates text one token at a time based on the preceding text. To control the output length and ensure relevance, we impose a constraint of 30 new tokens generated for each inference. This limitation is complemented by training the models to produce stop sequences (e.g., unique sequences such as “###”) to mark the end of each response. This enables us to determine the correct cutoff point of a response, beyond which hallucinations may occur. The imposition of a limit on new words, combined with the incorporation of stop sequences, allows the models to maintain flexibility in the length and complexity of the generated data elements, while simultaneously ensuring that the responses are concise and relevant.

### Evaluation strategy

To evaluate the performance of the models, we compare the estimated element responses against the reference data element responses in the synoptic reports written by the pathologists. While one strategy for this comparison could be simple string matching, this leads to incorrect assessment. For example, the estimated response “*Invasive adenocarcinoma*” compared against a reference response of “*Adenocarcinoma is invasive*” will lead to a poor score if done by exact string matching. This necessitates a more robust strategy to evaluate the model’s estimated responses. We utilize the BERT F1 score^43^ to measure model performances. To obtain an accuracy that corresponds to human evaluation, we determine a threshold by comparing the BERT F1 scores against manual evaluation on a test set composed of 100 unstructured reports for each of the 22 data elements (see Fig. 7). We use a threshold of 0.85, i.e., a BERT F1 > 0.85 is needed for estimated responses to be considered correct.

## Supplementary Material

Supplementary

**Supplementary information** The online version contains supplementary material available at https://doi.org/10.1038/s44401-025-00013-8.

## Figures and Tables

**Table 1 |:** Summary of the 7774 Mayo Clinic cancer pathology reports dataset

Data Element	% Non-missing	No. of Patients		Age in Years Mean [Min-Max]	Type of Cancer (%)							
		M	F		Breast	Lung	Pancreas	Skin	Digestive Organs	Female Reproductive Organs	Male Reproductive Organs	Other
Protocol Biopsy	25	1145	772	53.7 [19–85]	1	0	0	18	1	0	2	76
Procedure	70	2736	2682	60.6 [19–97]	13	6	3	2	13	8	16	38
Specimen	30	1096	1216	63.3 [21–97]	0	14	7	1	29	19	1	29
Primary Tumor	59	2401	2224	61.6 [19–97]	15	7	4	2	15	9	19	29
Specimen Integrity	17	530	801	63.3 [19–95]	0	23	0	5	4	30	3	34
Surgical Margins	44	1514	1898	62.1 [19–97]	20	10	5	3	20	7	2	33
Laterality	32	935	1545	58.0 [19–91]	29	13	0	1	1	0	1	54
Histologic Type	68	2674	2614	60.5 [19–97]	14	7	2	2	13	8	17	37
Histologic Grade	63	2525	2344	61.0 [19–97]	13	7	3	1	14	9	17	36
Mitotic Rate	13	209	793	59.3 [19–95]	59	1	4	7	3	0	0	26
Pathologic Staging Descriptors	30	1059	1295	60.9 [19–93]	25	6	5	2	14	8	15	25
Tumor Focality	27	873	1249	60.1 [19–92]	29	15	2	1	5	1	2	44
Tumor Site	43	1623	1698	60.5 [19–97]	7	10	5	3	19	8	1	47
Tumor Size	49	1584	2195	61.5 [19–97]	17	9	5	3	19	12	2	35
Lymphovascular Invasion	53	2226	1859	62.3 [19–97]	15	8	4	3	16	8	20	25
Regional Lymph Nodes	59	2376	2222	61.6 [19–97]	15	7	4	2	15	9	18	29
Lymph Node Sampling	30	917	1378	60.4 [19–93]	31	0	0	1	1	18	35	13
Number Examined	44	2270	1115	61.7 [19–97]	0	10	5	1	20	4	25	36
Number Involved	37	1957	924	62.1 [19–97]	0	10	6	1	22	3	26	32
Distant Metastasis	50	1940	1914	61.4 [19–97]	17	8	4	3	18	4	12	33
Perineural Invasion	16	764	462	63.1 [21–97]	0	2	13	4	47	0	1	32
Treatment Effect	29	1505	784	62.3 [19–95]	9	13	5	1	23	1	34	14

We show the top 22 most frequently reported data elements. The data elements listed do not occur in every report—the percent non-missing indicates the percentage of reports containing the corresponding data element.

## Data Availability

The data usage was approved by a Mayo Clinic Institute Review Board (IRB) and was determined to be exempt research. Due to Mayo Clinic policy and to protect privacy, the raw electronics health records data cannot be shared. The data presented in the figures and the code used to train the models is available here: https://github.com/bioIKEA/SynopsisGPT.

## Code availability

Due to Mayo Clinic policy and to protect privacy, the raw electronics health records data cannot be shared. The data presented in the figures and the code used to train the models is available here: https://github.com/bioIKEA/SynopsisGPT.
