# Supplementary material for: Synoptic reporting by summarizing cancer pathology reports using large language models
Source: Npj Health Syst. 2025 Apr 1;2:11. doi: 10.1038/s44401-025-00013-8 (PMC12858025; doi:10.1038/s44401-025-00013-8)
Supplement: Supplementary file 1 — Supplementary Information [file 44401_2025_13_MOESM1_ESM.pdf]

Synoptic Reporting by Summarizing Cancer Pathology Reports using Large Language Models

Sivaraman Rajaganapathy, Ph.D.[1], Shaika Chowdhury, Ph.D.[1], Xiaodi Li, Ph.D.[1], Vincent Buchner[1], Zhe He, Ph.D.[2], Rui Zhang, Ph.D.[3], Xiaoqian Jiang, Ph.D.[4], Ping Yang, M.D., Ph.D.[5], James R. Cerhan, M.D., Ph.D.[5], Nansu Zong, Ph.D.[1, *].

[1]*Department of Artificial Intelligence and Informatics, Mayo Clinic, Rochester, MN, USA;*

[2]*School of Information, Florida State University, Tallahassee, FL, USA;*

[3]*Division of Computational Health Sciences, University of Minnesota, Minneapolis, MN, USA;*

[4]*Department of Health Data Science and Artificial Intelligence, UTHealth, Houston, TX, USA;*

[5]*Department of Quantitative Health Sciences, Mayo Clinic, Rochester, MN, USA.*

*Corresponding author, Nansu Zong, Ph.D., Email: zong.nansu@mayo.edu

Supplementary Information

Supplementary Note 1: Components of the Pathology Report

Each pathology report in our data consists of four main components, which are:

1. Gross description: A *free text* record of a pathologist's observations of the tissue sample at the macroscopic level [1].
2. Preliminary frozen section consultation: A *free text* record of a pathologist's observations obtained through a special rapid analysis of a tissue sample that is frozen and reviewed during an ongoing surgery [27, 28].
3. Diagnosis: A *free text* record of the pathologist's definitive diagnosis that integrates knowledge from the gross description, the optional frozen section consultation, and microscopic analysis.
4. Synoptic report: A *structured* summary created from the gross description, the preliminary frozen section consultation, and the final diagnosis [4].

By *free text,* we refer to reports that are written in a natural language format, which sometimes exhibit regularity in format (e.g. section headers, bulleted lists etc.), but are not uniformly organized using a standard schema.

Supplementary Note 2: Identification of Classification Type and NLP Data Elements

Supplementary Table 1: Strategy for identifying classification type and NLP type data elements. The table shows the results from analysis of the entirety of our reference synoptic reports that have been written by physicians. The 'Number of Unique Responses' indicates the total number of unique element responses in the reference synoptic reports observed for each data element in all the data. The column 'Number of Synoptic Reports' shows the number of synoptic reports which contain the data element response (i.e. number of non-missing reports). The 'Ratio (%) – Unique Responses/Synoptic Reports' represents the ratio computed by dividing the number of unique responses (column 2) by the corresponding number of synoptic reports (column 3) expressed as a percentage. We consider that data elements whose ratios of unique responses per synoptic report is less than 5% as classification type. That is, we can group the element responses to a finite closed set without significant loss of meaning and information. The data elements selected as classification type are shown in **bold**. We consider the data elements *'Number Involved'* and *'Number Examined'* as NLP type since these are numeric. The column 'Number of Labels' shows the number of unique labels chosen after condensing semantically similar element responses to one unique label.

Data Element	Number of Unique Responses	Number of Synoptic Reports	Ratio (%) - Unique Responses/Synoptic Reports	Number of Labels (after Compression)	Data Element Category
**Regional Lymph Nodes**	**82**	**4629**	**1.8**	**6**	**Classification**
**Distant Metastasis**	**72**	**3889**	**1.9**	**3**	**Classification**
**Lymphovascular Invasion**	**89**	**4102**	**2.2**	**5**	**Classification**
**Perineural Invasion**	**29**	**1229**	**2.4**	**4**	**Classification**
**Primary Tumor**	**121**	**4656**	**2.6**	**7**	**Classification**
**Laterality**	**78**	**2518**	**3.1**	**6**	**Classification**
Number Involved	106	2898	3.7	NA	NLP
**Pathologic Staging Descriptors**	**87**	**2369**	**3.7**	**6**	**Classification**
Number Examined	142	3415	4.2	NA	NLP
**Specimen Integrity**	**58**	**1343**	**4.3**	**4**	**Classification**
Protocol Biopsy	122	1957	6.2	NA	NLP
Tumor Focality	140	2132	6.6	NA	NLP
Histologic Grade	436	4922	8.9	NA	NLP
Lymph Node Sampling	228	2299	9.9	NA	NLP
Treatment Effect	262	2304	11.4	NA	NLP
Procedure	900	5486	16.4	NA	NLP
Histologic Type	995	5353	18.6	NA	NLP
Mitotic Rate	205	1017	20.2	NA	NLP
Surgical Margins	977	3441	28.4	NA	NLP
Specimen	730	2327	31.4	NA	NLP
Tumor Site	1259	3379	37.3	NA	NLP
Tumor Size	3140	3813	82.3	NA	NLP

Supplementary Note 3: Generative Models Used

We selected three classes of models for our study, the Bidirectional Encoder Representations from Transformers (BERT) [5], the Generative Pre-trained Transformer 2 (GPT-2) [6], the Large Language Model Meta AI-2 (LLAMA-2) [7], and the Large Language Model Meta AI-3 (LLAMA-3) [8]. The exact variants of the model class used, and their relevant features are summarized in **Supplementary Table 2**. The number of parameters roughly correspond to the size and complexity of the model. The maximum input length is the limit on the size of a block of text a model can process. This input length is measured in tokens, where 1 token is approximately 4 characters in length. All the models we use are *pre-trained,* wherein the models have been previously trained on a large corpus of publicly available, non-specialized textual data [5–7]. We use the offline, fully accessible versions of these models offered through the Hugging Face Transformers library in Python [9].

Supplementary Table 2: A summary of the model classes used in this study and their relevant features. Model variant refers to the exact variant of the model used from a selection available. The number of parameters represents the complexity and size of the model. The maximum input length is the maximum number of tokens (1 token is approximately 4 characters) in an input text the model can process at a time. The training data is the dataset that was used to pre-train the model.

Model Class	Model Variant Used	Number of Parameters	Maximum Input Length (tokens)	Data Used for Pre-Training
BERT	BERT large model (uncased)	334M	512	Dataset of 11,038 unpublished books and English Wikipedia [5].
GPT-2	GPT-2	124M	1024	A proprietary dataset created by mining the web for heuristically determined high quality text [6].
LLAMA-2	LLAMA-2-7b	7000M	4096	A proprietary dataset of 2 trillion tokens created from publicly available sources without sources rich in personal and private information [7].
LLAMA-3	LLAMA-3-8b	8000M	8192	A proprietary dataset from various sources collected till end of 2023 without including domains that contain personally identifiable information [8].

Supplementary Note 4: Overview of alternative approaches for automatic cancer synoptic reporting

Supplementary Table 3: An overview of studies on automatic cancer synoptic reporting (excluding LLM based approaches). Direct experimental comparison with our fine-tuned LLMs is possible only when either the model and model weights are provided by the authors, or training is possible either using our data or author provided annotated data.

Study	Study Data; *(Data Elements)*	Number of Data Elements	Applicability			Model Available?	Training Possible?	Comparable Experimentally?	Performance Reported
			Classification Type Data	NLP Type Data	Unstructured Reports				
Schadow and McDonald, 2003 [10]	275 Semi-structured pathology reports. *(Acquired Abnormality, Anatomical Abnormality, Body System, Body Location or Region, Biomedical or Dental Material, Body Part, Body Space or Junction, Cell or Molecular Dysfunction, Diagnostic Procedure, Disease or Syndrome, Embryonic Structure, Finding, Neoplastic Process, Organ, Pathologic Function, Substance, Sign or Symptom, Spatial Concept, Tissue, Therapeutic or Preventive Procedure).*	20	Yes	Yes	No	No	No	No	91% accuracy (manual semantic match).
Wu et al., 2020 [11]	3,632 TCGA pathology reports with annotations. *(Cancer Type, Subtype, Laterality, Histologic Grade, TNM Stage, Diagnosis.)*	6	Yes	No	Yes	No	No	No	0.67-0.94 Macro F1.
**Alawad et al., 2020** [12]	71233 unstructured cancer pathology reports. *(Site, Laterality, Behavior, Histology, Grade.)*	5	Yes	No	Yes	No	Yes	Yes	0.55-0.96 Macro F1.
Lam et al., 2022 [13]	678 pathology reports. *(Dysplasia Grade, Dysplasia Location, Adenocarcinoma, Gleason Grades: Primary, Secondary, Total, Tissue with Carcinoma, Amount of Gleason 4/5, Amount of Carcinoma.)*	9	Yes	Yes	No	No	No	No	0.86-1.00 Cohen's κ.
**Zhou et al., 2022** [14]	4,543,184 clinical notes and 1,278,805 pathology reports (pre-training). 50 clinical notes and 200 pathology reports with annotations (fine-tuning). *(Hormone Receptor Type, Hormone Receptor Status, Tumor Size, Tumor Site, Cancer Grade, Histological Type, Tumor Laterality, Cancer Stage.)*	8	Yes	Yes	Yes	Yes	No	Yes	0.876 Macro F1.
Zeng et al. 2023 [15]	1438 pathology reports. *(Cancer Grade, Cancer Subtype, Lesion Position.)*	3	Yes	Yes	Yes	No	No	No	0.916 Micro F1.

Experimental comparison with MT-CNN

To train the MT-CNN model, the authors use a multi-task learning technique to categorize free-text cancer pathology report to simultaneously provide responses for 5 different data elements [12]. The MT-CNN trained using the hard parameter sharing approach was shown to have superior classification performance for a wide range of cancers [12]. The authors of the MT-CNN model are unable to share the original training data and the model weights to comply with data privacy regulations. However, the code for the model architecture and training is available to the public. The MT-CNN takes a report level classification approach, which is applicable only for classification type data elements. Further, the authors condense the unique responses for each closed data element selected to be between 3 to 65 unique labels. We have identified the classification and NLP type data elements in our data in **Supplementary Table 1**. We then condensed the number of unique element responses by assigning semantically identical response the same label. **Supplementary Table 1** also shows the number of unique labels allocated for the classification type data elements. Due to this label set compression and to provide a fair comparison in favor of the baseline model, we use balanced accuracy to compare the performance with our best performing fine-tuned LLM. Note: using the BERT F1 score for the MT-CNN model, where the semantic similarity of the MT-CNN label is compared with reference element response in the physician provided synoptic report leads to lower scores for MT-CNN due to the label compression.

Experimental comparison with CancerBERT

The CancerBERT model is a cancer domain-specific model that identifies named entities within the text of unstructured pathology reports [14]. The authors focus on 8 named entities, which are 8 data elements describing cancer phenotypes. We are able to compare experimentally, 6 of the 8 data elements that are common in our datasets. We obtained a copy of the CancerBERT model along with the model weights and use the model as-is in adapting it for automatic synoptic reporting. The CancerBERT model uses NER, wherein, the model annotates each word in the unstructured pathologic report with a label. The label identifies the word as belonging to one of the 8 breast cancer phenotypes or none of them. The 8 labels provided by the model are *hormone receptor type, hormone receptor status, tumor size, tumor site, cancer grade, histologic type, tumor laterality, and cancer stage.* Since we do not have the equivalent of *hormone receptor type* and *hormone receptor status* in our dataset, we chose to use the latter 6 data elements to compare the performance with our best performing fine-tuned LLM. We note that the NER task performed by the CancerBERT model is an intermediate step in automatic synoptic reporting. This is because for each report, the CancerBERT model generates multiple element responses for each data element under consideration. We chose to make the comparison in favor of the CancerBERT model by considering the CancerBERT response that leads to the maximum of the BERT F1 score. Fine-tuning the CancerBERT model on our data requires expert annotation of our pathologic reports at the word level. This fine-grained expert annotation is infeasible and beyond the scope of our project.

REFERENCES

1 Geller SA, Horowitz RE. Gross examination. *Methods Mol Biol*. 2014;1180:3–19. doi: 10.1007/978-1-4939-1050-2_1

2 WILSON LB. A METHOD FOR THE RAPID PREPARATION OF FRESH TISSUES FOR THE MICROSCOPE. *Journal of the American Medical Association*. 1905;XLV:1737. doi: 10.1001/jama.1905.52510230037003c

3 Black C, Marotti J, Zarovnaya E, *et al.* Critical evaluation of frozen section margins in head and neck cancer resections. *Cancer*. 2006;107:2792–800. doi: 10.1002/cncr.22347

4 Cancer Protocol Templates. College of American Pathologists. https://www.cap.org/protocols-and-guidelines/cancer-reporting-tools/cancer-protocol-templates (accessed 22 March 2024)

5 Devlin J, Chang M-W, Lee K, *et al.* BERT: Pre-training of Deep Bidirectional Transformers for Language Understanding. 2019.

6 Radford A, Wu J, Child R, *et al.* Language Models are Unsupervised Multitask Learners.

7 Touvron H, Martin L, Stone K, *et al.* Llama 2: Open Foundation and Fine-Tuned Chat Models. 2023.

8 Dubey A, Jauhri A, Pandey A, *et al.* The Llama 3 Herd of Models. 2024.

9 Wolf T, Debut L, Sanh V, *et al.* HuggingFace's Transformers: State-of-the-art Natural Language Processing. 2020.

10 Schadow G, McDonald CJ. Extracting Structured Information from Free Text Pathology Reports.

11 Wu J, Tang K, Zhang H, *et al.* Structured Information Extraction of Pathology Reports with Attention-based Graph Convolutional Network. *2020 IEEE International Conference on Bioinformatics and Biomedicine (BIBM)*. 2020:2395–402.

12 Mohammed A, Gao S, Qiu JX, *et al.* Automatic extraction of cancer registry reportable information from free-text pathology reports using multitask convolutional neural networks. *JAMIA*. 2020;27:89–98.

13 Lam H, Nguyen F, Wang X, *et al.* An accessible, efficient, and accurate natural language processing method for extracting diagnostic data from pathology reports. *J Pathol Inform*. 2022;13:100154. doi: 10.1016/j.jpi.2022.100154

14 Zhou S, Wang N, Wang L, *et al.* CancerBERT: a cancer domain-specific language model for extracting breast cancer phenotypes from electronic health records. *Journal of the American Medical Informatics Association*. 2022;29:1208–16.

15 Zeng KG, Dutt T, Witowski J, *et al.* Improving Information Extraction from Pathology Reports using Named Entity Recognition. *Res Sq*. 2023;rs.3.rs-3035772. doi: 10.21203/rs.3.rs-3035772/v1
